# Neural dynamics implement a flexible decision bound with a fixed firing rate for choice: a model-based hypothesis

**DOI:** 10.3389/fnins.2014.00318

**Published:** 2014-10-21

**Authors:** Dominic Standage, Da-Hui Wang, Gunnar Blohm

**Affiliations:** ^1^Department of Biomedical and Molecular Sciences, Queen's UniversityKingston, ON, Canada; ^2^Department of Systems Science/National Key Laboratory of Cognitive Neuroscience and Learning, Beijing Normal UniversityBeijing, China

**Keywords:** speed-accuracy trade-off, neural dynamics, bounded integration, decision threshold, threshold-baseline difference

## Abstract

Decisions are faster and less accurate when conditions favor speed, and are slower and more accurate when they favor accuracy. This speed-accuracy trade-off (SAT) can be explained by the principles of bounded integration, where noisy evidence is integrated until it reaches a bound. Higher bounds reduce the impact of noise by increasing integration times, supporting higher accuracy (*vice versa* for speed). These computations are hypothesized to be implemented by feedback inhibition between neural populations selective for the decision alternatives, each of which corresponds to an attractor in the space of network states. Since decision-correlated neural activity typically reaches a fixed rate at the time of commitment to a choice, it has been hypothesized that the neural implementation of the bound is fixed, and that the SAT is supported by a common input to the populations integrating evidence. According to this hypothesis, a stronger common input reduces the difference between a baseline firing rate and a threshold rate for enacting a choice. In simulations of a two-choice decision task, we use a reduced version of a biophysically-based network model (Wong and Wang, 2006) to show that a common input can control the SAT, but that changes to the threshold-baseline difference are epiphenomenal. Rather, the SAT is controlled by changes to network dynamics. A stronger common input decreases the model's effective time constant of integration and changes the shape of the attractor landscape, so the initial state is in a more error-prone position. Thus, a stronger common input reduces decision time and lowers accuracy. The change in dynamics also renders firing rates higher under speed conditions at the time that an ideal observer can make a decision from network activity. The difference between this rate and the baseline rate is actually greater under speed conditions than accuracy conditions, suggesting that the bound is not implemented by firing rates *per se*.

## 1. Introduction

In decision making experiments, subjects make faster, less accurate decisions when conditions favor speed, and make slower, more accurate decisions when conditions favor accuracy (e.g., Bogacz et al., 2010a; Heitz and Schall, 2012). These data describe the speed-accuracy trade-off (SAT) and can be explained by the principles of bounded integration. According to these principles, noisy evidence for the alternatives of a decision is integrated until the running total for one of the alternatives reaches a criterion level. The running total is referred to as a decision variable and the criterion is referred to as the bound. A higher bound allows evidence to be integrated for longer, increasing the percentage of correct decisions. A lower bound has the opposite effect. These abstract models have been invaluable in characterizing the computations underlying decisions and the SAT (see Smith and Ratcliff, 2004; Ratcliff and McKoon, 2008; Bogacz et al., 2010b).

The computations characterized by bounded integration models are hypothesized to be implemented by competitive interactions between neural populations selective for the alternatives of a decision (Usher and McClelland, 2001; Wang, 2002; Machens et al., 2005; Bogacz et al., 2006; Wong and Wang, 2006; Standage et al., 2011; You and Wang, 2013). According to this widely held hypothesis, temporal integration and competitive interactions are supported by recurrent excitation and feedback inhibition respectively, where each population implements a decision variable and a choice is made when the aggregate firing rate of one of the populations reaches a threshold. This hypothesis is supported by electrophysiological recordings from several cortical areas in non-human primates performing decision tasks, where the spike rates of neurons responsive to the chosen alternative (target-in neurons) increase over several hundreds of milliseconds prior to the animal's choice, and the spike rates of neurons unresponsive to the chosen alternative (target-out neurons) are much lower (e.g., Roitman and Shadlen, 2002; Thomas and Pare, 2007; Bollimunta and Ditterich, 2011; Ding and Gold, 2012).

Under several task paradigms, target-in activity of putative integrator neurons has been shown to reach an approximately fixed rate at the time of commitment to a choice (the choice threshold), regardless of the speed or accuracy of decisions (Hanes and Schall, 1996; Shadlen and Newsome, 2001; Roitman and Shadlen, 2002; Churchland et al., 2008; Purcell et al., 2010; Ding and Gold, 2012). These data have been interpreted as indicating that the neural implementation of the bound is fixed across conditions emphasizing speed over accuracy or *vice versa* (see Bogacz et al., 2010b). Under the assumption of linear integration, adjusting the starting point of a decision variable is equivalent to adjusting the bound, so it has been hypothesized that subjects trade speed and accuracy by adjusting the “baseline” rate of integrator populations, i.e., the activity on which a decision variable builds (see Bogacz et al., 2010b). According to this hypothesis, the SAT is controlled by a cognitive signal projecting uniformly to all integrator populations, where a stronger (weaker) signal favors speed (accuracy) by decreasing (increasing) the difference between the choice threshold and baseline activity (the threshold-baseline difference). We refer to this possibility as the threshold-baseline hypothesis (a.k.a. the changing-baseline hypothesis, Bogacz et al., 2010b). Several recent neuroimaging (Forstmann et al., 2008; Ivanoff et al., 2008; van Veen et al., 2008; Wenzlaff et al., 2011) and electrophysiological (Heitz and Schall, 2012; Hanks et al., 2014) studies have provided evidence for such a signal, reporting higher baseline (pre-stimulus) activity in decision-correlated cortical areas under speed conditions than accuracy and/or neutral conditions.

Here, we present an alternative hypothesis that does not assume linear integration. As above, we assume that a cognitive signal controls the SAT by projecting uniformly to integrator populations, but the underlying mechanism is grounded in the framework of attractor dynamics (e.g., Machens et al., 2005; Bogacz et al., 2006; Wong and Wang, 2006; Standage et al., 2011; You and Wang, 2013; see Wang, 2008, 2012 for review). According to this framework, integration times are determined by the non-linear dynamics of decision circuitry, where stronger and weaker dynamics furnish shorter and longer integration times respectively (Wong and Wang, 2006; Standage et al., 2011). The SAT can therefore be accomplished by any mechanism that modulates the strength of dynamics within and between neural populations selective for the decision alternatives (see Standage et al., 2014). Spatially non-selective excitation provides just such a mechanism (Salinas and Abbott, 1996), where a stronger (weaker) signal corresponds to speed (accuracy) conditions (Furman and Wang, 2008; Roxin and Ledberg, 2008). Of course, this input also entails higher (lower) baseline activity under speed (accuracy) conditions. In attractor network models, higher (lower) baseline activity will indeed decrease (increase) the threshold-baseline difference, but this decrease (increase) is epiphenomenal. The SAT is supported by the resulting changes to network dynamics.

Below, we use a neurally-derived model (Wong and Wang, 2006) to demonstrate that adjusting the strength of spatially non-selective excitation can control the SAT (Furman and Wang, 2008; Roxin and Ledberg, 2008). We demonstrate that this signal raises (lowers) the baseline activity of integrator populations, consistent with higher (lower) baseline activity under speed (accuracy, neutral) conditions in SAT experiments (Forstmann et al., 2008; Ivanoff et al., 2008; van Veen et al., 2008; Wenzlaff et al., 2011; Heitz and Schall, 2012; Hanks et al., 2014). We use a fixed choice threshold in the model, so the spatially non-selective signal decreases (increases) the threshold-baseline difference under speed (accuracy) conditions, relative to a neutral condition. We demonstrate that the threshold-baseline difference cannot account for the SAT in the model, since raising (lowering) the threshold to compensate for the higher (lower) baseline activity under the speed (accuracy) condition does not “untrade” speed and accuracy, i.e., reinstating the threshold-baseline difference of the neutral condition does not recover the neutral behavior of the model. Using dynamic systems analysis, we show that a higher (lower) baseline decreases (increases) the effective time constant of integration of the network under speed (accuracy) conditions, accounting for the SAT in a manner consistent with a flexible bound, while also changing the shape of the decision space so as to further decrease (increase) accuracy. Finally, we show that decision-selective firing rates in the model are actually higher (lower) under speed (accuracy) conditions at the time at which an ideal observer can discriminate between the rates of the integrator populations; as is the difference between these rates and the baseline rate (the discrimination-baseline difference). Thus, the discrimination-baseline difference increases under speed conditions and decreases under accuracy conditions, opposite to the principles of the threshold-baseline hypothesis. Our analysis explains these observations.

Our simulations show that under the framework of attractor dynamics, there is no discrepancy between a flexible bound and a fixed choice threshold. The bound—or the difference between the bound and the starting point of a decision variable—is a computational device for controlling the duration of evidence accumulation in abstract models. It can be implemented by the effective time constant of integration of decision circuitry, with corresponding changes to the decision space. This space and its time evolution are emergent properties of network dynamics and are qualitatively different than the synaptic current required to elicit choice behavior.

## 2. A common input to integrators controls the SAT in an attractor model, but not by the threshold-baseline difference

**Figure 1:**
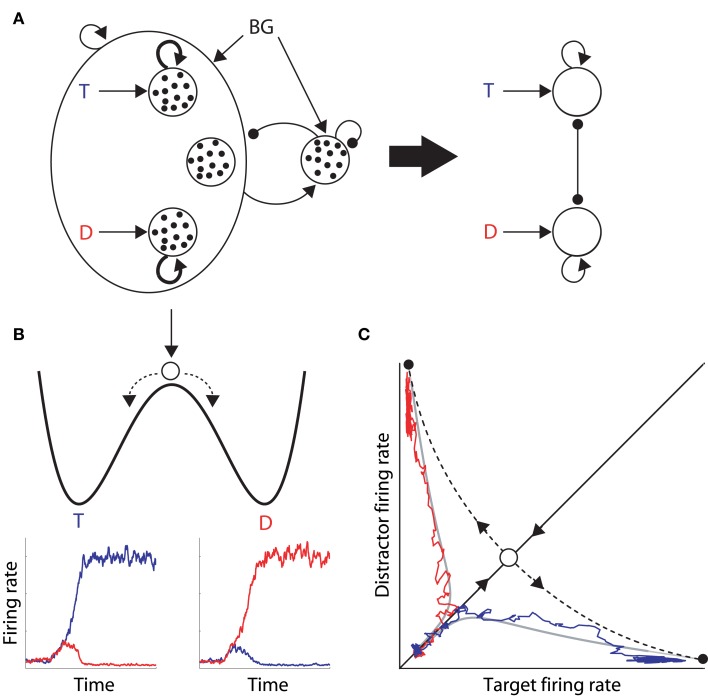
**(A)** The reduced model by Wong and Wang (2006), approximating a biophysically-based cortical network model (left of the thick arrow) with a 2-variable system (right). The thick arrow depicts the derivation of the latter from the former. The large oval on the left depicts a network of cortical pyramidal neurons. Inside the oval, the three open circles depict the target and distractor populations with selective input T and D respectively, and a population unresponsive to the evidence for either alternative. Looping arcs depict recurrent synapses, which are stronger within each selective population (thicker arcs). All pyramidal neurons excite a common inhibitory pool, which uniformly inhibits all pyramidal neurons. Excitatory and inhibitory synapses are depicted by arrows and closed circles respectively, small black dots depict individual neurons, and BG refers to background input. **(B)** Cartoon depiction of an attractor “energy landscape” for 2-choice decisions, where the energy decreases over time. An unstable steady state (high energy) separates two stable attractors (low energy), corresponding to the target and distractor stimuli. Conceptually, a ball placed between the two attractors will eventually role one way or the other, depicted by the dashed arrows. The ball enters an attractor basin sooner (later) under speed (accuracy) conditions because the dynamics evolve more quickly (slowly). Below the cartoon, the firing rates of target (blue) and distractor (red) neural populations are plotted over time during two decision trials, corresponding to the ball rolling into the target attractor basin (left) and the distractor attractor basin (right). **(C)** Decision space for two choices. Stable (solid) and unstable (dashed) manifolds of the saddle point (intersection of the manifolds, see text). The system moves toward this state along the stable manifold and is repelled along the unstable manifold. The firing rates of the winning populations in the two decision trials in **(B)** are plotted against each other, superimposed on the decision space, along with two noise-free trajectories (gray) with initial conditions inside each attractor basin. On each trial, the network state moves along the stable manifold before being repelled toward an attractor.

In their seminal study, Wong and Wang (2006) used analytic methods to reduce a biophysically-based cortical network model (Wang, 2002) to a 2-variable system, tractable for analysis (depicted in Figure 1A). They showed that each of the populations selective for the decision alternatives corresponds to a stable state in the space of possible states of network activity, i.e., each population corresponds to an attractor (Figures 1B,C). The attractors are separated by an unstable “saddle” steady state with two manifolds: a stable manifold that draws the network toward the saddle point, and an unstable manifold that repels it toward one of the stable attractors (Figure 1C). They further calculated the time constants of these two manifolds, showing that the dynamics in the vicinity of the saddle support integration times much longer than the time constants of decay of contributing biophysical processes, such as those of neurons and synapses.

We used Wong and Wang's (2006) model in simulations of a 2-choice random dot motion (RDM) task (Supplementary Material Section 1). We ran 1000 trials for each motion coherence *c* ∈ {0, 1, 2, 4, 8, 16, 32}%, where the motion stimulus was provided for 5s following a 2.5s pre-stimulus interval. We refer to the integrator population receiving the stronger (weaker) stimulus as the target (distractor) population. We modeled speed and accuracy conditions by increasing and decreasing a uniform input to the two populations respectively, relative to a neutral condition. To this end, we adjusted the mean background current *I*_0_, capturing the total input current from upstream neurons other than those encoding motion stimuli. This current therefore subsumes the hypothesized cognitive signal controlling the SAT. Because the model's parameter values and corresponding dynamics are rigorously described by Wong and Wang (2006), we used the same parameter values here (excepting *I*_0_ and its corresponding standard deviation, see Supplementary Material Section 1).

**Figure 2:**
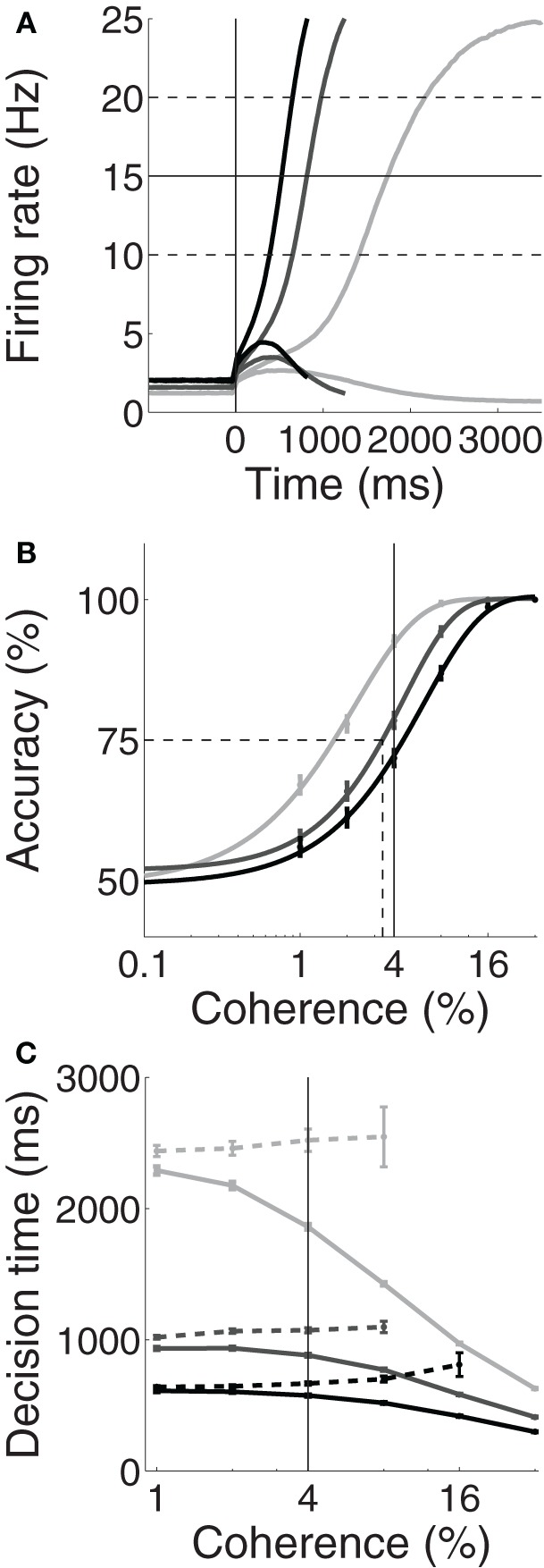
**Trading speed and accuracy as a function spatially non-selective input *I*_0_**. Simulated neural activity **(A)** and resulting psychometric **(B)** and chronometric **(C)** curves for neutral (*I*_0_ = 321pA, medium gray), speed (*I*_0_ = 325pA, black) and accuracy (*I*_0_ = 316pA, light gray) conditions. **(A)** Trial-averaged firing rates for coherence *c* = 4%. For each condition, the upper and lower curves show the mean rate over all correct trials for the target and distractor populations respectively. The vertical line at 0ms indicates the time of simulated motion onset. To the left of this line, pre-stimulus/baseline firing rates are higher (lower) under speed (accuracy) conditions compared to the neutral condition. Thus, the threshold-baseline difference is smaller (larger) under speed (accuracy) conditions. The solid horizontal line shows the “default” choice threshold θ = 15Hz used by Wong and Wang (2006). The dashed horizontal lines depict other possible thresholds. **(B)** The percentage of correct trials as a function of coherence. The data are fitted with a Weibull function for each condition. Error bars show standard error. The solid vertical line indicates coherence *c* = 4%, corresponding to the firing rates in **(A)**. The dotted lines indicate the coherence value at 75% accuracy (see Figure 3A). **(C)** Mean decision times over coherence for correct (solid) and error (dashed) trials for each condition. Error bars show standard error. The vertical line indicates coherence *c* = 4%, corresponding to the firing rates in **(A)**.

Unsurprisingly, the spatially non-selective current *I*_0_ produced higher and lower pre-stimulus (baseline) firing rates under speed and accuracy conditions respectively, compared to the neutral condition. Baseline rates can be seen to the left of the vertical line in Figure 2A for an example coherence value (*c* = 4%, see Figure caption). The resulting SAT can be seen in Figures 2B,C, where the psychometric curve is shifted to the right and left under speed and accuracy conditions respectively; and for correct and error trials, mean decisions times are shorter and longer respectively. Thus, Figure 2 shows that by raising and lowering baseline activity, uniform input to both integrator populations controls the SAT. At first glance, these results appear to support the threshold-baseline hypothesis.

**Figure 3:**
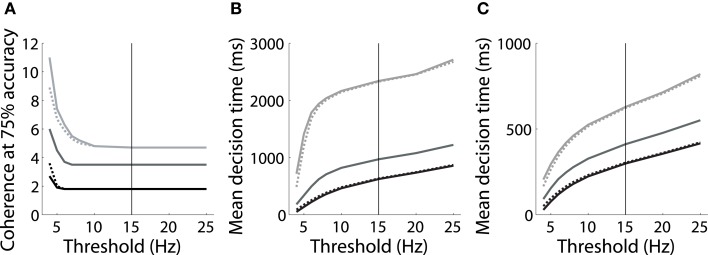
**(A)** The value of simulated motion coherence at which accuracy equals 75% for a range of choice thresholds under speed (black), neutral (medium gray) and accuracy (light gray) conditions (see dashed lines in Figure 2B). Dotted curves show results for simulations in which the threshold was raised (speed condition) and lowered (accuracy condition) by Δ_*ns*_ and Δ_*na*_ respectively (see text). Solid curves show results for the corresponding unadjusted threshold. Adjusting the choice threshold makes little difference to accuracy, i.e., the solid and dotted curves are similar for speed and accuracy conditions, and do not overlay the neutral curve. **(B,C)** Mean decision times over all trials for coherence *c* = 1% **(B)** and *c* = 32% **(C)**. Conventions are the same as in **(A)**. Adjusting the threshold by Δ_*ns*_ and Δ_*na*_ makes little difference to decision times, regardless of the threshold chosen. The solid vertical line in each panel indicates the threshold used by Wong and Wang (2006).

However, the threshold-baseline hypothesis dictates that the speed and accuracy of decisions are determined by the threshold-baseline difference. According to this hypothesis, a fixed threshold-baseline difference will produce uniform decision making performance, regardless of the rate of baseline activity. The threshold-baseline hypothesis therefore requires that any changes to the speed or accuracy of decisions resulting from a change in baseline activity (with a fixed threshold) can be “reversed” by an equal change to the threshold. We therefore increased the threshold under the speed condition by the difference between baseline activity under speed and neutral conditions (Δ_*ns*_, the mean difference over the last 1000 ms of the pre-stimulus interval), and we decreased the threshold under the accuracy condition by the difference between baseline activity under neutral and accuracy conditions (Δ_*na*_). These adjustments to the threshold did not recover the psychometric and chronometric curves produced under the neutral condition, i.e., the black and light gray curves in Figures 2B,C do not overlay the medium gray curves. Denoting the threshold used by Wong and Wang (2006) as θ (vertical line in Figure 3), increasing (decreasing) θ by Δ_*ns*_ (Δ_*na*_) under the speed (accuracy) condition has almost no effect on performance. The same is true for any value of the choice threshold above θ. For thresholds below θ, the effect of these adjustments increases with decreasing threshold, but the psychometric (Figure 3A) and chronometric (Figures 3B,C) curves under speed and accuracy conditions do not come close to overlaying the neutral curves. For the lowest value of the threshold, there is a moderate effect on the psychmetric curves (the difference between the solid and dotted curves for speed and accuracy conditions), but such a low threshold does not allow a firing-rate excursion, so this moderate effect can only be achieved if the model deviates from the neural data on which the threshold-baseline hypothesis is founded, i.e., a fixed rate of target-in activity that is much higher than target-out activity at the time of commitment to a choice (e.g., Shadlen and Newsome, 2001; Roitman and Shadlen, 2002; Thomas and Pare, 2007; Purcell et al., 2010; Bollimunta and Ditterich, 2011; Ding and Gold, 2012). See the Discussion for other issues with such a low threshold. The psychometric and chronometric curves break down for thresholds lower than those in the figure. Note that Figures 3B,C show results for coherence values of *c* = 1% and *c* = 32% respectively. Values in between these extremes yield the same qualitative result. These results demonstrate that the threshold-baseline hypothesis does not account for the SAT under the principles of the attractor framework.

## 3. The SAT is controlled by network dynamics

**Figure 4:**
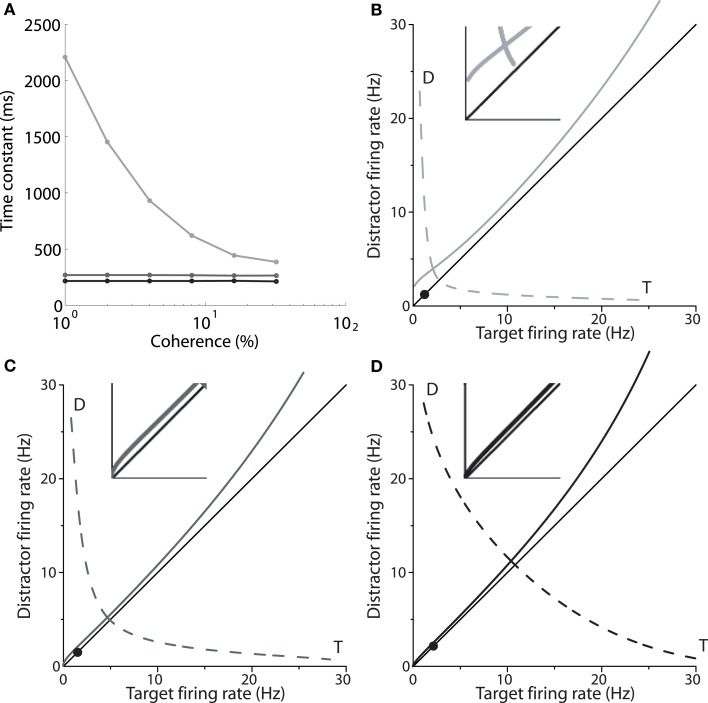
**(A)** The time constant of the unstable manifold of the saddle point (see Figure 1) for speed (black), neutral (medium gray) and accuracy (light gray) conditions, as a function of coherence. The time constant determines the time over which the system is repelled from the saddle toward an attractor corresponding to the target or the distractor (Figure 1C; T and D in **B–D**). **(B–D)** Stable (solid) and unstable (dashed) manifolds of the saddle for the accuracy (**B**, *I*_0_ = 316pA), neutral (**C**, *I*_0_ = 321pA) and speed (**D**, *I*_0_ = 325pA) conditions for coherence *c* = 4%. At low rates below the saddle, the stable manifold is pushed closer to the midline with increasing *I*_0_, while rates at the saddle increase. Solid circles show the initial state of the network. Insets show close-ups of the stable manifold and the midline at frequencies ≤5 Hz.

Returning to Figure 2A, the mean firing rates following motion onset (to the right of the vertical line) point to the mechanism by which the spatially non-selective input *I*_0_ controls the SAT in the model. The rate of increase of target activity is higher and lower under speed and accuracy conditions respectively, relative to the neutral condition. The different rates of increase reflect the dynamics furnished by the different values of *I*_0_ under speed, accuracy and neutral conditions. As shown by Wong and Wang (2006), the dynamics in the vicinity of the saddle point determine the length of time the network can integrate evidence, which can be approximated by the time constant of the unstable manifold (the effective time constant of integration, Supplementary Material Section 2). Wong and Wang (2006) calculated this time constant for several values of the strength of recurrent excitation, showing the consequent changes to the speed and accuracy of decisions (see their Figure 11). Figure 4A shows these calculations for our changes to *I*_0_. Under speed and accuracy conditions, higher and lower values of *I*_0_ furnish shorter and longer time constants respectively, relative to the neutral condition. Here, it is worth noting that the effective time constant behaves in exactly the same way as the bound of bounded integration models, decreasing (increasing) integration time under speed (accuracy) conditions (Figure 4A). Additionally, the shape of the attractor landscape changes with *I*_0_. Figures 4B–D show that for a given task difficulty (*c* = 4% in the figure), higher values of *I*_0_ push the stable manifold toward the midline at low rates below the saddle point. Since the network approaches the saddle from below (Figure 1C) and since errors occur when noise pushes the state of the network over the stable manifold (Wong and Wang, 2006), this re-positioning of the stable manifold further lowers (raises) accuracy under speed (accuracy) conditions. This mechanism is evident in Figures 4B–D, in which the solid circle in each panel shows the mean initial state of the network (immediately prior to the onset of evidence). With increasing *I*_0_, the stable manifold moves toward this initial state, which becomes increasingly precarious. Thus, a common input to integrators controls the rate of baseline activity, but the SAT does not result from the consequent changes to the threshold-baseline difference. The SAT results from the changes to network dynamics.

**Figure 5:**
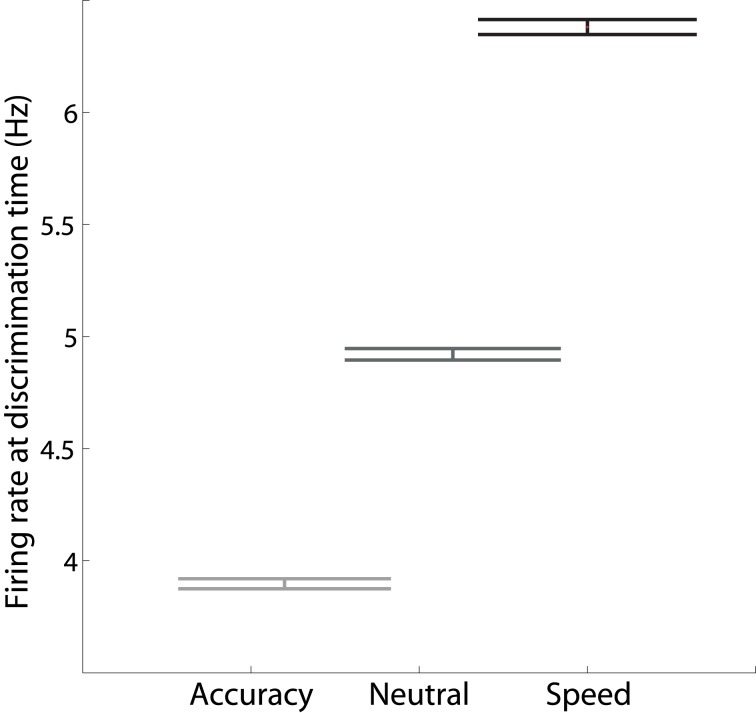
**The mean firing rate of the target population at the time at which an ideal observer can discriminate target activity from distractor activity, calculated across all positive coherence values**. The rate is higher (lower) under speed (accuracy) conditions.

Increasing *I*_0_ not only re-positions the stable manifold, but also re-positions the saddle point, so that both populations fire at higher rates (Figures 4B–D). This change in position of the saddle dictates that firing rates will be higher when the network begins its descent into an attractor basin under speed conditions. In other words, firing rates will be higher when decision-selective rates separate from those of the competing population. To confirm this effect, we used signal detection theory to determine when an ideal observer can discriminate target activity from distractor activity in the model under speed, accuracy and neutral conditions (Supplementary Material Section 3). Signal detection theory is commonly used to estimate the time of target selection from neural data (Thompson et al., 1996; Cohen et al., 2009) and assumes that a downstream circuit makes decisions by discriminating the activity of neural populations selective for the alternatives (see Standage and Pare, 2011). Firing rates at the time of discrimination were higher under speed conditions and lower under accuracy conditions (Figure 5).

Next, we subtracted the baseline rate under speed, accuracy and neutral conditions from the corresponding rate at discrimination time (the discrimination-baseline difference). The discrimination-baseline difference was larger under speed conditions and smaller under accuracy conditions. Because decisions are over when the firing rates separate, the rate at this time approximates a “decision threshold,” as opposed to the choice threshold (see the Discussion). To summarize: the difference between this decision threshold and baseline activity is larger under speed conditions and smaller under accuracy conditions in the model. Thus, stronger (weaker) non-selective input under speed (accuracy) conditions modulates decision-selective firing rates in a manner opposite to the principles of the threshold-baseline hypothesis. We confirmed these findings with an alternative method, in which decision times (and correctness) were determined by the last intersection of target and distractor activity on each trial, i.e., decisions were made when target and distractor activity separated for the final time. The mean rate at the time of separation was higher (lower) under speed (accuracy) conditions, as was the difference between this rate and the baseline rate (not shown). Importantly, our analysis in this section makes two predictions for electrophysiological studies of the SAT: (1) target-in and target-out data will separate at higher (lower) rates under speed (accuracy) conditions, and (2) the discrimination-baseline difference will be larger (smaller) under speed (accuracy) conditions.

## 4. Discussion and conclusions

We have demonstrated that spatially non-selective excitation can control the SAT in an attractor model (Figures 2B,C), as shown previously (Furman and Wang, 2008; Roxin and Ledberg, 2008). The non-selective input increases and decreases baseline activity under speed and accuracy conditions respectively (Figure 2A), which unavoidably decreases and increases the difference between baseline activity and a fixed choice threshold. The threshold-baseline difference, however, does not control the SAT in the model (Figure 3). Rather, an increase (decrease) in non-selective input increases (decreases) the strength of network dynamics, which decreases (increases) the effective time constant of integration (Figure 4A) and renders the initial state of the network closer to (farther from) the stable manifold of the saddle, the crossing of which results in errors (Figures 4B–D).

Our findings are consistent with the hypothesis that a cognitive signal controls the SAT by adjusting a uniform input to integrator populations (see Bogacz et al., 2010b; Standage et al., 2014). This hypothesis is supported by neuroimaging (Forstmann et al., 2008; Ivanoff et al., 2008; van Veen et al., 2008; Wenzlaff et al., 2011) and electrophysiological (Heitz and Schall, 2012; Hanks et al., 2014) data from SAT tasks, where pre-stimulus activation has been shown to be higher (lower) under speed (accuracy, neutral) conditions. Like the threshold-baseline hypothesis, our results are consistent with these data. Our results conflict with the threshold-baseline hypothesis because the changes in network dynamics engendered by a uniform input dwarf the corresponding changes to the threshold-baseline difference. A related reason is that the choice threshold is qualitatively different than the bound of bounded integration models. The rate of target-in activity at the time of commitment to a choice has been shown to be considerably higher than the rate at which this activity separates from target-out activity (see e.g., Shadlen and Newsome, 2001; Roitman and Shadlen, 2002; Bollimunta and Ditterich, 2011; Ding and Gold, 2012). Under the framework of attractor dynamics, this excursion of target-in activity corresponds to the repulsion of a decision network from the saddle along its unstable manifold. Thus, these neural data suggest that the choice threshold is much higher than the saddle. As such, changes to the choice threshold will not influence decision accuracy over a broad range, unlike the bound of bounded integration models. This effect is clear in Figure 2A. As noted in Section 3, the rate at which target and distractor activity separates can be thought of as a “decision threshold,” but our simulations predict that this rate is not fixed across speed and accuracy conditions. Indeed, we predict that it changes (Figure 5) in a manner opposite to a flexible bound (e.g., Ratcliff and McKoon, 2008; Bogacz et al., 2010a). Our findings therefore suggest that the bound is not implemented in terms of firing rates *per se*. In this regard, the astute reader may have noticed our use of the term “choice threshold” when referring to decision-selective firing rates at the time of commitment to a choice, as opposed to the more conventional “decision threshold.” We believe the latter term is misleading in this context.

There are potential advantages to choice thresholds being higher than decision thresholds. For example, a high choice threshold alleviates the need for fine tuning (Roxin and Ledberg, 2008). Furthermore, the difference between the choice threshold and a decision threshold provides a buffer between decisions and their enactment. This buffer may confer advantages to decision makers. For instance, a high choice threshold gives an upstream decision variable the opportunity to suppress its competitors, that is, the choice is not made until the “winning” integrator population is firing at a high rate and the losing populations are firing at much lower rates. Thresholds are hypothesized to be implemented by networks with very strong dynamics (Simen, 2012), which are poorly suited to decision making (Standage and Pare, 2011), i.e., they implement an all-or-none response to a critical level of input. If the respective rates of the choice threshold and the decision threshold were similar (a small buffer), then the difference between the decision variables would be smaller when the largest one reaches the choice threshold, increasing the possibility that the thresholding circuit would inadvertently choose the wrong decision variable. Simultaneous electrophysiological recordings from decision circuitry and thresholding circuitry would be informative in this regard. It seems unlikely that target-in activity in one structure would coincide with target-out activity in the other, even infrequently. Another possibility is that thresholding circuitry implements an ideal observer of integrator circuitry, where back-projections from the former to the latter account for the excursion of decision-selective activity prior to choice selection (see Simen, 2012). Under this scenario, bidirectionally-coupled decision circuits would collectively implement both integration and choice, a compelling possibility that warrants further investigation.

Another perspective on the difficulties of equating the difference between the bound and the starting point of a decision variable with the threshold-baseline difference relates to levels of abstraction in models of brain function (Marr, 1982; Trappenberg, 2010). From this perspective, bounded integration models can be considered algorithms that characterize the computations underlying decisions. They have been (and continue to be) invaluable for our understanding of decision processing and the SAT, but it is not necessary to attribute direct biological correlates to each of their parameters. Qualitatively, the effective time constant of integration under speed and accuracy conditions changes in the same manner as the bound (Figure 4A) and therefore provides a plausible neural implementation of this abstract term, but the corresponding changes to the attractor basins show that this interpretation may be overly simplistic (Figures 4B–D). Note that we do not suggest the twain shall never meet. Far from it, formal equivalence has been shown between different classes of (linear) bounded integration models and the (non-linear) biophysically-based model on which our simulations are based (Bogacz et al., 2006). The constraints under which these models are equivalent define the relationship between decision models at these two levels of abstraction, allowing the systematic consideration of one class in terms of the other. Where earlier work has largely considered the commonalities between classes of model, e.g., the range of parameters under which non-linear, feedback-inhibition models are well-approximated by linear integration models (Usher and McClelland, 2001; Bogacz et al., 2006), we have focused on their differences. In this sense, we have shown what is lost in translation in relation to the SAT, suggesting that caution is warranted when interpreting neural data in terms of models that are purposefully simplified. Note that earlier discussions of the threshold-baseline hypothesis have made it clear that changes to the bound and the starting point of a decision variable are not equivalent in all abstract models (Bogacz et al., 2010b). For more extensive treatment of the constraints of the threshold-baseline hypothesis in relation to implementation-level models, see Marshall et al. (2012).

It is possible that a different kind of threshold-baseline difference could account for the SAT. If the baseline rate of *thresholding circuitry* were increased (decreased) under speed (accuracy) conditions, then lower rates of integrator activity would be sufficient to elicit choice behavior, i.e., to drive the relevant motor circuitry (see Standage et al., 2014 for review). As such, a cognitive signal controlling the SAT could bypass integrator populations. However, the rates of integrator populations at the time of commitment to a choice would be lower under speed conditions and higher under accuracy conditions, which conflicts with recent electrophysiological recordings from putative integrator neurons showing the opposite profile of activity (Heitz and Schall, 2012). Notably, these data also show higher (lower) baseline rates and a higher (lower) rate of increase under speed (accuracy) conditions, suggesting that speed and accuracy conditions do modulate integrator neurons. These findings are qualitatively reproduced by our simulations (Figure 2A).

Finally, we do not suggest that single-circuit attractor models provide a complete picture of decision making. For example, these models produce slower mean decision times on error trials than correct trials because the network state has to cross the unstable manifold (Wong and Wang, 2006; Standage et al., 2011), but error trials are faster than correct trials under some task paradigms (see Smith and Ratcliff, 2004). Such shortcomings point to the need for coupled-circuit models (e.g., Lo and Wang, 2006; Standage et al., 2013). The recent surge in neuroimaging studies of decision making and the SAT represents an important direction in this regard, identifying contributing brain regions and pointing to their respective roles in decision processing (Forstmann et al., 2008; Ivanoff et al., 2008; van Veen et al., 2008; Forstmann et al., 2010; van Maanen et al., 2011; Wenzlaff et al., 2011; Green et al., 2012; Ho et al., 2012). Guided by these data, models of distributed decision circuitry are an exciting direction in decision neuroscience (Frank, 2006; Lo and Wang, 2006; Bogacz and Gurney, 2007). Simulations of the bidirectional coupling between circuits supporting evidence integration and choice may be highly informative about the relationship between decision bounds and choice thresholds.

### Conflict of interest statement

The authors declare that the research was conducted in the absence of any commercial or financial relationships that could be construed as a potential conflict of interest.
