# Supplementary material for: Neural dynamics implement a flexible decision bound with a fixed firing rate for choice: a model-based hypothesis
Source: Front Neurosci. 2014 Oct 21;8:318. doi: 10.3389/fnins.2014.00318 (PMC4204603; doi:10.3389/fnins.2014.00318)
Supplement: Supplementary file 1 [file Presentation1.PDF]

Neural dynamics implement a flexible decision bound with a fixed firing rate for choice: a model-based hypothesis
Supplementary Material

Dominic Standage, Da-Hui Wang and Gunnar Blohm

1 The reduced biophysical network model

For accuracy conditions, I_0 was given the lowest value for which the network made a decision on at least 95% of trials across all values of motion coherence used, tested in increments of 1pA. In other words, I_0 was given the lowest value that consistently supported decision dynamics (Figure 1, main text). For speed conditions, I_0 was given the highest value that did not destabilize the background state, *i.e.* the highest value that did not support competitive dynamics prior to motion onset. For the neutral condition, we chose a middle value. As we varied I_0, we adjusted its standard deviation σ_{noise} to maintain the same I_0/σ_{noise} ratio, though this adjustment did not influence our results.

We used the simplest version of the model by Wong and Wang (2006), as presented in their Appendix:

$$
\begin{aligned}
&\frac{dS_i}{dt} = -\frac{S_i}{\tau_{NMDA}} + (1 - S_i)\gamma H_i \\
&H_i = \frac{ax_i - b}{1 - exp(-d(ax_i - b))} \\
&x_1 = J_{11}S_1 - J_{12}S_2 + I_0 + I_1 + I_{noise,1} \\
&x_2 = J_{22}S_2 - J_{21}S_1 + I_0 + I_2 + I_{noise,2} \\
&I_i = J_{A,ext}\mu_0(1 \pm \frac{c}{100\%}) \\
&\tau_{AMPA}\frac{dI_{noise,i}(t)}{dt} = -I_{noise,i}(t) + \eta_i(t)\sqrt{\tau_{AMPA}\sigma^2_{noise}}.
\end{aligned} \tag{1}
$$

Index i refers to the two neural populations, selective for the decision alternatives. The state variable S refers to the activation of NMDA receptors at recurrent synapses onto pyramidal neurons, hypothesized to dominate local-circuit cortical processing (Wang, 2002; Wong & Wang, 2006). H is the input-output (transfer, gain) function, transforming current to firing rate, with parameters $a = 270(\text{VnC})^{-1}$, $b = 108$Hz and $d = 0.154$s. $\gamma = 0.641$ is a scale factor. The time constants of decay of NMDA receptor and AMPA receptor activation are $\tau_{NMDA} = 100$ms and $\tau_{AMPA} = 2$ms respectively. The synaptic couplings strengths are $J_{11} = J_{22} = 0.2609$nS, $J_{12} = J_{21} = 0.0497$nA and $J_{ext} = 0.00052\text{nA}\cdot\text{Hz}^{-1}$. The total input current I is comprised of the motion stimulus with firing rate $\mu_0 = 30$Hz, and the spatially non-selective current $I_0 = [0.316, 0.321, 0.325]$nA for accuracy, neutral and speed conditions respectively. η is a normally distributed random variable with mean I_0 and standard deviation $\sigma_{noise} = I_0/16.275$, maintaining the same ratio of I_0 to σ_{noise} used by Wong and Wang (2006). See their study for the derivation of these parameters. All simulations were run in Matlab (version R2012A), using the standard implementation of Euler's forward method with a time step of 0.1ms.

2 Dynamic systems analysis

To calculate the time constant of the unstable manifold of the saddle point, let

$$\frac{dS_1}{dt} = f_1(S_1, S_2)$$
$$\frac{dS_2}{dt} = f_2(S_1, S_2), \tag{2}$$

where S_1 and S_2 are defined in Section 1 above. The steady states of the model satisfy the equations

$$f_1(\bar{S_1}, \bar{S_2}) = 0$$
$$f_2(\bar{S_1}, \bar{S_2}) = 0. \tag{3}$$

The Jacobian matrix of the steady states is

$$\mathbf{J} = \begin{pmatrix} \frac{\partial f_1}{\partial S_1} & \frac{\partial f_1}{\partial S_2} \\ \\ \frac{\partial f_2}{\partial S_1} & \frac{\partial f_2}{\partial S_2} \end{pmatrix}_{(\bar{S_1},\bar{S_2})} \tag{4}$$

and the corresponding eigenvalue λ can be calculated by solving $|\mathbf{I}\lambda - \mathbf{J}| = 0$. If a steady state is a saddle point, one of the eigenvalues is positive and the other is negative. The time constant is the inverse of the positive eigenvalue.

3 Ideal observer analysis

We used signal detection theory (Green & Swets, 1966) to determine how well an ideal observer could discriminate target activity from distractor activity in the model, estimating the separation of the distributions of target and distractor activity at successive 1 ms intervals. To this end, we calculated receiver operating characteristic curves (ROC) from the mean rates of the target and distractor populations over all trials and positive coherence values. The area under the ROC (AUROC) quantifies the separation of their distributions (see Thompson, Hanes, Bichot, and Schall (1996)). We quantified the probability of neuronal discrimination by a least squares fit of the AUROCs to a Weibull function

$$w(t) = z - (z - y) \cdot exp(-(t/p)q), \tag{5}$$

where t is the time after motion stimulus onset, p is the time at which the function reaches 63% of its maximum, q is the slope parameter, and z and y are the upper and lower limits of the function respectively. The time at

which the fitted function reached 75% of its maximum was considered to be discrimination time (Thompson et al., 1996; Standage & Pare, 2011).

References

Green, D. M., & Swets, J. A. (1966). *Signal detection theory and psychophysics.* New York: Wiley.

Standage, D., & Pare, M. (2011). Persistent storage capability impairs decision making in a biophysical network model. *Neural Networks*, *24*, 1062–1073.

Thompson, K., Hanes, D., Bichot, N., & Schall, J. (1996). Perceptual and motor processing stages identified in the activity of macaque frontal eye field. *Journal of Neurophysiology*, *76*, 440–455.

Wang, X.-J. (2002). Probabilistic decision making by slow reverberation in cortical circuits. *Neuron*, *36*, 955–968.

Wong, K.-F., & Wang, X.-J. (2006). A recurrent network mechanism of time integration in perceptual decisions. *The Journal of Neuroscience*, *26*, 1314–1328.
